# The effects of pre versus post workout supplementation of creatine monohydrate on body composition and strength

**DOI:** 10.1186/1550-2783-10-36

**Published:** 2013-08-06

**Authors:** Jose Antonio, Victoria Ciccone

**Affiliations:** 1 Exercise and Sports Sciences, Nova Southeastern University, 3532 S. University Drive, University Park Plaza Suite 3532, Davie, FL 33314, USA

**Keywords:** Creatine, Supplement timing, Body composition, Fat free mass, Dietary supplement

## Abstract

**Background:**

Chronic supplementation with creatine monohydrate has been shown to promote increases in total intramuscular creatine, phosphocreatine, skeletal muscle mass, lean body mass and muscle fiber size. Furthermore, there is robust evidence that muscular strength and power will also increase after supplementing with creatine. However, it is not known if the timing of creatine supplementation will affect the adaptive response to exercise. Thus, the purpose of this investigation was to determine the difference between pre versus post exercise supplementation of creatine on measures of body composition and strength.

**Methods:**

Nineteen healthy recreational male bodybuilders (mean ± SD; age: 23.1 ± 2.9; height: 166.0 ± 23.2 cm; weight: 80.18 ± 10.43 kg) participated in this study. Subjects were randomly assigned to one of the following groups: PRE-SUPP or POST-SUPP workout supplementation of creatine (5 grams). The PRE-SUPP group consumed 5 grams of creatine immediately before exercise. On the other hand, the POST-SUPP group consumed 5 grams immediately after exercise. Subjects trained on average five days per week for four weeks. Subjects consumed the supplement on the two non-training days at their convenience. Subjects performed a periodized, split-routine, bodybuilding workout five days per week (Chest-shoulders-triceps; Back-biceps, Legs, etc.). Body composition (Bod Pod®) and 1-RM bench press (BP) were determined. Diet logs were collected and analyzed (one random day per week; four total days analyzed).

**Results:**

2x2 ANOVA results - There was a significant time effect for fat-free mass (FFM) (F = 19.9; p = 0.001) and BP (F = 18.9; p < 0.001), however, fat mass (FM) and body weight did not reach significance. While there were trends, no significant interactions were found. However, using magnitude-based inference, supplementation with creatine post workout is possibly more beneficial in comparison to pre workout supplementation with regards to FFM, FM and 1-RM BP. The mean change in the PRE-SUPP and POST-SUPP groups for body weight (BW kg), FFM (kg), FM (kg) and 1-RM bench press (kg) were as follows, respectively: Mean ± SD; BW: 0.4 ± 2.2 vs. 0.8 ± 0.9; FFM: 0.9 ± 1.8 vs. 2.0 ± 1.2; FM: -0.1 ± 2.0 vs. −1.2 ± 1.6; Bench Press 1-RM: 6.6 ± 8.2 vs. 7.6 ± 6.1.

Qualitative inference represents the likelihood that the true value will have the observed magnitude. Furthermore, there were no differences in caloric or macronutrient intake between the groups.

**Conclusions:**

Creatine supplementation plus resistance exercise increases fat-free mass and strength. Based on the magnitude inferences it appears that consuming creatine immediately post-workout is superior to pre-workout vis a vis body composition and strength.

## Introduction

Chronic supplementation with creatine has been shown to increase lean body mass and enhance exercise performance [1-10]. Creatine supplementation for as brief a period as 3 days has been shown to produce a significant increase in skeletal muscle volume and exercise performance according to Ziegenfuss et al. [9]. One week of supplementation has been shown to increase body weight 1.4 kg (range 0.00 to 2.7 kg) [11]. Furthermore, creatine supplementation combined with resistance training resulted in a 6.3% increase in body weight and fat-free mass after a 12 week treatment period [12]. Subjects with initially low levels of intramuscular creatine (e.g. vegetarians) are more responsive to supplementation than those who regularly consume meat [13]. However, not all investigations demonstrate a positive effect of creatine supplementation vis a vis body composition [14-18].

It has not yet been fully elucidated what effect nutrient timing (i.e. consuming nutrients pre, during and/or post workout) has on the adaptive response to exercise [19-24]. However, based on the preponderance of evidence, it is apparent that consuming the proper nutrients during the periworkout time period may enhance lean body mass and expedite skeletal muscle recovery [25-29]. For instance, Tipton et al. demonstrated that consuming an essential amino acid solution pre workout resulted in a greater net muscle protein synthesis than that when the solution is consumed after exercise; this increase in muscle protein synthesis is believed to be the result of an increased delivery of amino acids to the leg [29]. Cribb and Hayes discovered that consuming a protein-carbohydrate-creatine supplement immediately pre and post workout resulted in greater gains in lean body mass, muscle fiber size and muscular strength in comparison to morning and evening consumption [25]. It is apparent that the timing of nutrient intake does indeed affect the adaptive response to exercise but it is not known if there is a difference between pre versus post workout consumption of a supplement or nutrient combination. Therefore, the purpose of this investigation was to determine if there was a difference in pre versus post workout supplementation of creatine on body composition and muscular strength.

## Methods

### Subjects

Nineteen male recreational bodybuilders (mean ± SD: age, 23.1 ± 2.9 years; height, 166.0 ± 23.2 cm; body weight, 80.2 ± 10.4 kg) completed this study. Participants were otherwise healthy college-age students who had been resistance training regularly for over a year. Individuals who were currently consuming other workout supplements or ergogenic aids were instructed to immediately stop consumption and complete at least a four-week washout period before entering the study. All procedures involving human subjects were approved by Nova Southeastern University’s Human Subjects Institutional Review Board in accordance with the Helsinki Declaration, and written informed consent was obtained prior to participation.

### Experimental design

Subjects were randomly assigned to one of two groups: a PRE-SUPP or POST-SUPP group. The PRE-SUPP group consumed 5 grams of creatine monohydrate immediately prior to training. The POST-SUPP group consumed the same amount of creatine immediately after training. Following pre-testing data collection, participants began a periodized four-week resistance training program that was self-administered. On off-training days, subjects consumed creatine at their convenience. The total treatment duration was four weeks.

### Resistance training protocol

All subjects followed a periodized, split-routine bodybuilding training regimen geared primarily for skeletal muscle hypertrophy. The participants trained 5 days a week for 4 weeks for a total of 20 training sessions. Each training session lasted approximately 60 minutes. The program was as follows:

Week 1

Mon – Chest, Shoulder, Triceps (3 × 15, sets × reps; does not include 2 warm up sets)

Tues – Hips, Legs (3 × 15)

Wed – Back, Biceps (3 × 15)

Thurs – Chest, Shoulders, Triceps (3 × 10)

Fri – Hips, Legs (3 × 10)

Sat – REST

Sun - REST

Week 2

Mon – Back, Biceps (3 × 10)

Tues – Chest, Shoulder, Triceps (3 × 5)

Wed – Hips, Legs (3 × 5)

Thurs – Back, Biceps (3 × 5)

Fri – Chest, Shoulder, Triceps (3 × 15)

Sat – REST

Sun – REST

Week 3

Mon – Hips, Legs (3 × 15)

Tues – Back, Biceps (3 × 15)

Wed – Chest, Shoulders, Triceps (3 × 10)

Thurs – Hips, Legs (3 × 10)

Fri – Back, Biceps (3 × 10)

Sat – REST

Sun – REST

Week 4

Mon – Chest, Shoulders, Triceps (3 × 5)

Tues – Hips, Legs (3 × 5)

Wed – Back, Biceps (3 × 5)

Thurs – Chest, Shoulder, Triceps (3 × 15)

Fri – Hip, Legs (3 × 15)

Sat – REST

Sun – REST

### Choice of exercises for each body part split

Chest – (subject performed 3 of these) flat bench press, incline bench press, cable cross-overs, pec deck, flat bench flies, decline bench press; Shoulders (subject performed 3 of these) – upright row, machine military press, dumbbell overhead presses, lateral dumbbell raises, shoulder shrugs; Triceps (subject performed two of these) – triceps pushdowns, dips, French press; Back (subject performed four of these) – Wide grip lat pulldown, narrow grip lat pulldown, chin ups, cable rows, dumbbell rows, dumbbell flies; Biceps (subject performed three of these) – standing barbell curls, standing EZ bar curl, concentration curls, preacher curls, hammer curls; Legs/Hips (subject performed five of these) – Back squats, Smith machine squats, Leg Press, Lunges, Leg curls, Leg extensions, calf raise (seated or standing), Stiff-legged deadlift. On test days, participants reported to NSU’s Exercise and Sports Science lab after a 3 hour fast and refrained from participating in vigorous activity or exercise in the 24-hour period prior to testing. Subjects were asked to maintain their normal dietary intake for the duration of the study and to refrain from ingesting any other dietary supplement that may enhance body composition (e.g. protein, amino acids, etc.).

### Food diary, workout log, body composition

Subjects provided a 24-hour diet recall on one random day on week 1, 2, 3, and 4 (as determined by the investigators). Dietary intake was measured using the Nutribase® program. Height was measured using standard anthropometry and total body weight was measured using a calibrated scale. Body composition was assessed by whole body densitometry using air displacement via the Bod Pod® (COSMED USA, Concord, CA). All testing was performed in accordance with the manufacturer’s instructions. Briefly, subjects were tested while wearing only tight fitting clothing (swimsuit or undergarments) and an acrylic swim cap. The subjects wore the exact same clothing for all testing. Thoracic gas volume was estimated for all subjects using a predictive equation integral to the Bod Pod® software. The calculated value for body density used the Siri equation to estimate body composition. Data from the Bod Pod® included body weight, % body fat, fat free mass and fat mass. All testing was done with each subject at the same time of day (plus or minus 1 hour). Also, all subjects were required to keep a workout log showing the exercises with reps and sets performed.

### Exercise performance assessment

Subjects performed a 1 repetition maximum lifts (1-RM) on the bench press. Subjects warmed up (2 sets of 8–10 repetitions at approximately 50% of anticipated maximum) on the bench press. Subjects performed successive 1-RM lifts starting at about 70% of anticipated 1-RM and increased it by 5–10 lbs until the reaching a 1-RM. There was a two minute rest interval between sets. Each subject was allowed a maximum of three attempts.

### Statistical analysis

Data were analyzed utilizing five separate 2-way [group (Pre-Treatment [aka PRE-SUPP] vs. Post-Treatment [aka POST-SUPP]) × time (pre vs. post)] Analysis of Variance (ANOVA). When appropriate, follow-up analysis included paired sample *t*-test. An alpha level was set at p ≤ 0.05, and all analyses were performed using PASW version 18.0 (SPSS, Inc., Chicago, IL). The effects of nutrient timing plus resistance exercise were calculated as the changes from pretraining to post-training body composition and performance measurements among Pre-Treatment vs. Post-Treatment groups. Magnitude-based inferences were used to identify clinical differences in the measurement changes between the Pre-Treatment and Post-Treatment. Several studies have supported the use of magnitude-based inference statistics as a complementary tool for null hypothesis testing to reduce errors in interpretation and to provide more clinically meaningful results [30,31]. The precision of the magnitude inference was set at 90% confidence limits, using a p value derived from an independent *t*-test. Threshold values for positive and negative effect were calculated by multiplying standard deviations of baseline values by 20% [30]. Inferences on true differences between the exercise and control group were determined as positive, trivial, or negative according to methods previously described by Batterham and Hopkins [31]. Inferences were based on the confidence interval range relative to the smallest clinically meaningful effect to be positive, trivial, or negative. Unclear results are reported if the observed confidence interval overlaps both positive and negative values. The probability of the effect was evaluated according to the following scale: : <0.5%, most unlikely; 0.5-5%, very unlikely; 5-25%, unlikely; 25-75%, possibly; 75-95%, likely; 95–99.5%, very likely; >99.5%, most likely (Hopkins, 2010).

## Results

Twenty-two subjects were initially recruited for this investigation. Three subjects dropped out for no given reason. Nineteen healthy recreational male bodybuilders (age: 23.1 ± 2.9; height: 166.0 ± 23.2 cm; weight: 80.2 ± 10.4 kg) completed the study. There were no differences between groups for any of the baseline measures. 2×2 ANOVA results - There was a significant time effect for FFW (F = 19.9; p = 0.001) and BP (F = 18.9; p < 0.001), however FM and BW did not reach significance. While there were trends, no significant interactions were found (Table 1).

**Table 1:** Body composition and strength

		**Baseline**	**Post-test**	**Mean change**
**PRE-SUPP N = 9**	BW (kg)	82.5 ± 10.5	82.9 ± 10.6	0.4 ± 2.5
**POST-SUPP N = 10**		78.1 ± 10.4	78.9 ± 10.0	0.8 ± 0.9
**PRE-SUPP**	FFM (kg)	66.7 ± 6.9	67.6 ± 7.6	0.9 ± 1.8
**POST-SUPP**		65.9 ± 8.0	67.9 ± 8.6	2.0 ± 1.2
**PRE-SUPP**	FM (kg)	15.4 ± 4.9	15.3 ± 5.5	−0.1 ± 2.0
**POST-SUPP**		13.00 ± 4.0	11.8 ± 3.6	−1.2 ± 1.6
**PRE-SUPP**	% Body Fat	18.4 ± 4.1	18.2 ± 5.1	−0.2 ± 2.2
**POST-SUPP**		16.9 ± 4.8	15.0 ± 4.7	−1.9 ± 2.3
**PRE-SUPP**	1-RM BP	96.7 ± 21.9	103.3 ± 19.5	6.6 ± 8.2
**POST-SUPP**		103.2 ± 24.0	110.9 ± 25.4	7.7 ± 6.2

Values are mean ± SD. *1-RM* one repetition maximum, *BP* Bench Press, *BW* body weight, *FFM* fat-free mass, *FM* fat mass.

**Figure 1:**
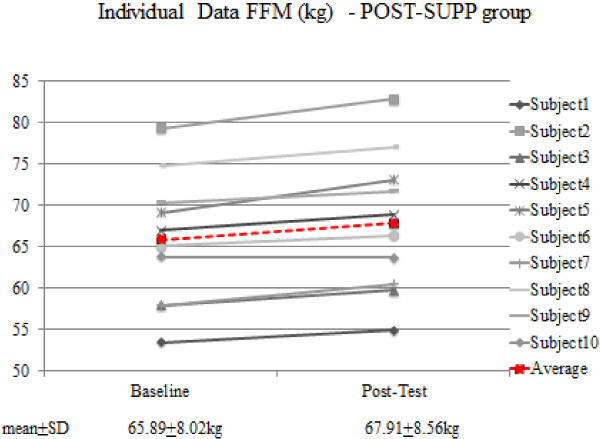
Individual data for FFM in the POST-SUPP group.

**Figure 2:**
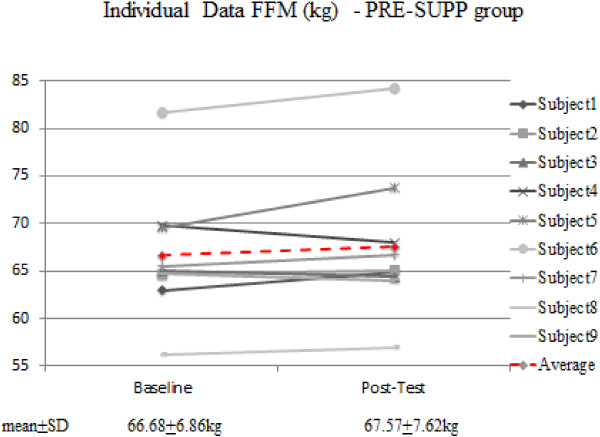
Individual data for FFM in the PRE-SUPP group.

Thus, using magnitude-based inference, supplementation with creatine post-workout is possibly more beneficial in comparison to pre-workout supplementation with regards to FFM, FM (Table 2, Figure 1, Figure 2) and 1-RM BP. It is apparent that everyone in the POST-SUPP group improved vis a vis FFM; however, this was not the case with the PRE-SUPP group (Figures 1 and 2).

**Table 2:** Magnitude-based inference results

	**POST-SUPP**	**PRE-SUPP**		
**Measures**	**Mean ± SD**	**Mean ± SD**	**Difference ± 90CI**^**a**^	**Qualitative Inference**
**BW (kg)**	0.8 ± 0.9	0.4 ± 2.2	0.4 ± 1.3	Trivial
**FFM (kg)**	2.0 ± 1.2	0.9 ± 1.8	1.1 ± 1.2	Possibly beneficial
**FM (kg)**	−1.2 ± 1.6	−0.1 ± 2.0	1.1 ± 1.5	Possibly beneficial
**1-RM BP (kg)**	7.6 ± 6.2	6.6 ± 8.2	1.2 ± 1.7	Likely beneficial

Changes in body composition and performance in PRE-SUPP vs. POST-SUPP groups, and qualitative inferences about the effects on body composition and bench press strength.

Values reported as mean ± standard deviation (SD); *BW* body weight, *FFM* fat-free mass, *FM* fat mass. ^a^ ± 90% CI: add and subtract this number to the mean difference to obtain the 90% confidence intervals for the true difference. Qualitative inference represents the likelihood that the true value will have the observed magnitude.

### Dietary variables

The macronutrient intake for the PRE-SUPP and POST-SUPP groups are summarized in Table 3. There were no significant differences between the groups. On average, both groups consumed a diet of 39-40% carbohydrate, 26% protein, and 35% fat. Both groups consumed 1.9 grams of protein per kg body weight.

**Table 3:** Dietary intake

	**PRE-SUPP**	**POST-SUPP**
**Total kcals**	2416 ± 438	2575 ± 842
**CHO g**	229 ± 53	261 ± 120
**CHO kcal**	915 ± 213	1046 ± 479
**CHO %**	39 ± 11	40 ± 10
**PRO g**	159 ± 41	147 ± 41
**PRO kcal**	637 ± 165	590 ± 163
**PRO %**	26 ± 4	25 ± 7
**FAT g**	96 ± 39	104 ± 48
**FAT kcal**	863 ± 359	939 ± 433
**FAT %**	35 ± 10	35 ± 8

Values are mean ± SD; no significant differences for any of the variables.

*CHO* carbohydrate, *PRO* protein.

## Discussion

The results from this study suggest that consuming creatine monohydrate post exercise may be superior to consuming it pre exercise with regards to improving body composition (i.e. gains in FFM, loss of FM). This is the first investigation to demonstrate that the timing of creatine intake affects the adaptive response to exercise. When subjects were pooled together, the gains in fat-free mass and muscular strength in the current investigation were similar to others. Rugby union football players who supplemented daily with creatine monohydrate over an 8-week period decreased fat mass (−1.9 kg) and increased lean tissue (+1.2 kg). They also performed better in bench and leg press tests [15]. Older men (71 yrs) who consumed creatine increased lean tissue mass (+3.3 kg) and improved lower body strength as measured using a 1-RM [32]. Using a single-limb training model, men and women who supplemented with creatine after training of the arms increased their muscle thickness. Interestingly, males had a greater increase in lean tissue mass with creatine supplementation than females [4]. In elite male handball players, creatine supplementation for 32 days resulted in an increase in 1-RM bench press (8.30 vs. 5.29 kg; creatine versus control) [33]. These and other investigations indeed show that creatine supplementation in general has a significant anabolic and performance-enhancing effect [34,35] which is in agreement with the current investigation. Mechanistically, creatine supplementation has been shown to increase muscle fiber size, enhance myosin heavy chain protein synthesis, activate satellite cells as well as increase the concentrations of intramuscular ATP and PCr [6,7,12,36,37].

However, whether supplement timing has a role in the adaptive response vis a vis creatine has not been previously investigated. Certainly, the most important aspect of the current investigation is that post workout supplementation of creatine may indeed be superior to pre workout supplementation. Data on protein and amino acid supplementation indicate that indeed the pre, during and post workout window are important times to consume nutrients though some studies demonstrate a neutral effect [20-24,38]. One study examined the effects of a solution of whey protein consumed either immediately before exercise or immediately following exercise. They found no difference in amino acid uptake between the groups [18]. In six subjects (3 men, 3 women) that randomly consumed a treatment drink (6 g essential amino acids, 35 g sucrose) or a flavored placebo drink 1 hour or 3 hours after a bout of resistance exercise, investigators found no difference in the anabolic response whether the drink was consumed 1 hour or 3 hours post exercise [39]. Indeed, others have found that timed protein supplementation immediately before and after exercise does not further enhance muscle mass or strength in healthy elderly men who habitually consume adequate amounts of dietary protein [40]. Also, timed protein-supplement ingestion in resistance-trained athletes during a 10-week training program does not further enhance strength, power, or body-composition changes [41].

On the other hand, consuming an essential amino acid solution immediately before resistance exercise elevates muscle protein synthesis to a greater extent than when the solution is consumed after exercise. The investigators postulated that this may be due to an increased delivery of amino acids to the leg [29]. Clearly, issues related to blood flow would not be advantageous to the POST-SUPP group in the current study.

Another study investigated the importance of immediate (P0) or delayed (P2: 2 hours post exercise) intake of an oral protein supplement upon muscle hypertrophy and strength over a period of resistance training in elderly males. In response to training, the cross-sectional area of the quadriceps femoris muscle and mean fiber area increased in the P0 group, whereas no significant increase was observed in P2. These investigators found no difference in the glucose or insulin response at P0 or P2, thus, it is not likely that differences in the hormonal environment contributed to the difference in muscle mass gain. Thus, the early intake of an oral protein supplement after resistance training is important for skeletal muscle hypertrophy [42].

Perhaps the seminal study vis a vis nutrient timing compared taking a protein-carbohydrate-creatine supplement either immediately pre and post exercise (PRE-POST) or in the morning and evening (MOR-EVE). Indeed the PRE-POST group demonstrated a greater increase in lean body mass and 1-RM strength in two of three assessments. Furthermore, type II muscle fiber cross-sectional area was larger in the PRE-POST group as well as intramuscular concentrations of creatine and glycogen [25]. Data from this investigation showed the intramuscular creatine and glycogen concentrations were greater in the PRE-POST versus MOR-EVE groups. Thus, taking the exact same supplement (but timed pre and post exercise) is significantly better than consuming it in the morning and evening.

Our investigation did not involve the use of protein, carbohydrate or amino acids. Whether creatine uptake is truly sensitive to timed intake is not entirely known despite the superior gains in the POST-SUPP group. Moreover, it is entirely possible that the difference in body composition and muscular strength between the two groups was the result of a small sample size. One individual in the POST-SUPP and three individuals in the PRE-SUPP group experienced a minor reduction in FFM. With regards to 1-RM bench press performance, two subjects in the PRE-SUPP group showed either no change or a decline in strength; on the other hand, only one subject in the POST-SUPP group showed no change in strength. All other subjects experienced an increase in strength.

The use of recreational bodybuilders in the current investigation is advantageous because it is difficult for highly trained individuals to experience an increase in FFM or muscular strength in the time frame allotted for this study. Nonetheless, of the 19 subjects that completed the study, 16-21% were non-responders regarding muscular strength and FFM. It should be noted that the nutrient intake (kcals, carbohydrate, fat and protein) was similar between the groups. In fact, each group consumed a high protein diet (1.9 grams of protein per kg bw daily); thus, it is not likely that dietary factors caused the discrepancy in the adaptive response to creatine supplementation and resistance training. Nevertheless, another consideration to take into account would be that because these recreational bodybuilders were already consuming large quantities of protein, this could have affected the results (i.e. they could already have a high amount of creatine stored intramuscularly and this may have blunted the results).

In conclusion, post workout supplementation with creatine for a period of 4 weeks in recreational bodybuilders may produce superior gains in FFM and strength in comparison to pre workout supplementation. The major limitations of this study include the small sample size as well as the brief treatment duration. Future studies should investigate creatine supplementation using resistance trained individuals for a longer duration.

## Competing interests

Jose Antonio PhD was a former sports science consultant to VPX® Sports.

## Authors’ contributions

VC and JA contributed significantly to all aspects of this study. Both authors read and approved the final manuscript.
